# Effect of Surface-Etching Treatment, Glaze, and the Antagonist on Roughness of a Hybrid Ceramic after Two-Body Wear

**DOI:** 10.3390/ma15196870

**Published:** 2022-10-03

**Authors:** Manassés Tercio Vieira Grangeiro, Camila da Silva Rodrigues, Natália Rivoli Rossi, Jadson Mathyas Domingos da Silva, Nathalia de Carvalho Ramos, João Paulo Mendes Tribst, Lilian Costa Anami, Marco Antonio Bottino

**Affiliations:** Institute of Science and Technology, São Paulo State University (Unesp), São José dos Campos, São Paulo 12245-000, Brazil

**Keywords:** hybrid ceramic, wear, roughness, surface treatment

## Abstract

Stains and glaze are effective procedures for achieving an aesthetic smoothness on indirect restorations. Thus, the effect of surface-etching treatments previous to the stain layer and the glaze application on the occlusal and antagonist wear of a hybrid ceramic were evaluated against different antagonists. Disc-shaped samples were prepared from polymer-infiltrated ceramic network (PICN) blocks. The specimens were divided into eight groups, according to the surface-etching treatment and glaze application: P (polished specimens); PG (polishing plus glaze); E (hydrofluoric acid etching plus stain); EG (acid etching plus stain plus glaze); A (aluminum oxide sandblasting plus stain); AG (sandblasting plus stain plus glaze); S (self-etching primer plus stain); SG (self-etching primer plus stain plus glaze). Half of the samples were subjected to a wear simulation with a steatite antagonist, and the other half was tested using a PICN antagonist. The test parameters were: 15 N, 1.7 Hz, 6 mm of horizontal sliding, 5000 cycles. The discs and the antagonists’ masses were measured before and after the wear tests. The average roughness and spacing defects were evaluated. The etching treatment affected the surface and antagonist mass loss when tested against steatite. AG showed the highest mass loss. This influence was not detected when using the PICN antagonist. The glaze application after staining ensures a smoother surface and avoids antagonist wear.

## 1. Introduction

Computer-aided design/computer-aided manufacturing (CAD/CAM) technology has been applied in dental practice for the last 25 years. The use of CAD/CAM allows for more reliable processing, which leads to restorations with a low fracture rate, high aesthetics, easy repair, and great longevity [1,2,3].

Among CAD/CAM materials, the polymer-infiltrated ceramic network (PICN) has emerged as a porous feldspathic ceramic matrix infiltrated with a copolymer (UDMA and TEGDMA) [4,5]. The resulting hybrid material presents an elastic modulus in the range of human dentin (~30 GPa) and offers easy machinability. This material ranges between ceramics and composites, since its mechanical properties surpass the conventional resin composites [6,7], and it leads to less antagonist wear than glass ceramics [8].

It is well-known that the surface roughness of restorative materials is an important clinical parameter, since it affects the biofilm adherence [9], wear kinetics, color, and tactile perception of the patient [10]. The occlusal wear of ceramic restorations can modify the occlusion pattern of the patients, harm aesthetics [11], and decrease the occlusion vertical dimension, which limits clinical success [12]. Stain and glaze layers are applied over the ceramic prosthesis, aiming to achieve aesthetics and smoothness [13]. Thus, the quality of these finishing treatments is strongly associated with the antagonist wear. A previous study has reported that a glaze layer applied over ceramic surfaces can be maintained for over 12 years in function [14]. However, little information is available regarding the photo-cured glaze layer indicated for using over PICN.

Manual finishing, stains, and glaze application are the most effective procedures for achieving a smooth surface on indirect restorations [8,15]. The manufacturer indicates the following procedural sequence for finishing PICN restorations: polishing with rubber points, etching with hydrofluoric acid or sandblasting with aluminum oxide, stain application and photo polymerization, and glaze application followed by final photo polymerization. The use of hydrofluoric acid etching or sandblasting before the characterization is suggested to ensure the adhesion between stain and PICN in the face of chewing forces. Nonetheless, alternative surface treatments could lead to even better results. On the other hand, a glaze layer is applied after the stain to ensure smoothness and wear resistance, which consequently results in less harm to the antagonist over time.

In this context, this paper aims to understand the role of surface-etching treatments (hydrofluoric acid, self-etching primer, and sandblasting) before the stain layer and the glaze application on the occlusal and antagonist wear of PICN restorations. Moreover, the effect of these different finishing sequences on two types of antagonists (steatite and hybrid ceramic) are explored. The tested hypothesis was that the different etching procedures would not affect the occlusal or antagonist wear of PICN.

## 2. Materials and Methods

The materials used in the present study are described in Table 1.

### 2.1. Specimen Preparation and Characterization

PICN blocks (Vita Enamic, Vita Zahnfabrik, Bad Sackingen, Germany) were rounded into 12 mm Ø cylinders, which were sliced into 1.2 mm-thick discs using a diamond saw in a cutting machine (Isomet^®^ 1000, Precision Sectioning Saw, Buehler, Lake Bluff, IL, USA) (N = 160). The discs were polished with silicon carbide sandpapers (#400, #600, and #1200) under water-cooling in a polishing machine (EcoMet™/AutoMet™250, Buehler, Lake Bluff, USA). The top and bottom surfaces were polished for 20 s with #400 grit to remove irregularities. Then, the top surfaces were polished to #1200 grit. The discs were randomly divided into eight groups (*n* = 20), according to the top surface-etching treatment before the extrinsic characterization and the glaze application, as described in Table 2.

The control group (P) was subjected to no surface treatment after polishing. The PG group received a photo-cured glaze layer after polishing. The photo-cured glaze (Vita enamic^®^ glaze, Vita Zahnfabrik, Bad Sackingen, Germany) was applied with a brush, and the polymerization was performed with LED (Valo LED, Ultradent, South Jordan, UT, USA) for 30 s (3200 mW/cm^2^).

Specimens from the E and EG groups were subjected to 5% hydrofluoric acid etching for 60 s. Then, the specimens were cleaned with a 5-min ultrasonic bath in distilled water and air-dried. A ceramic primer (Vita adiva^®^ cera-etch, Vita Zahnfabrik, Bad Sackingen, Germany) was applied onto the etched surface and left to dry for 30 min. Then, a thin black stain layer (Vita enamic^®^ stain, Vita Zahnfabrik, Bad Sackingen, Germany) was applied (1:1 powder: liquid) with a brush. The stain layer was photocured with LED for 6 s. The EG specimens were finished with a photo-cured glaze layer.

The A and AG groups were sandblasted with 50 μm aluminum oxide (Al_2_O_3_) for 10 s at 1 bar. The specimens were cleaned with an ultrasonic bath for 5 min in distilled water and then air-dried. The ceramic primer was applied and, after 30 min, a thin stain layer was applied. The AG group received a stained layer followed by glazing.

Specimens from the S and SG groups were etched with a self-etching primer (Monobond^®^ Etch & Prime, Ivoclar Vivadent, Schaan, Liechtenstein). The primer was actively applied with a disposable brush for 20 s and left to react for 40 s. Then, the specimens were cleaned with an ultrasonic bath in distilled water for 5 min. After drying, a thin layer of stain was applied. The SG group also received a glaze layer.

### 2.2. Two-Body Abrasive Wear

**Figure 1:**
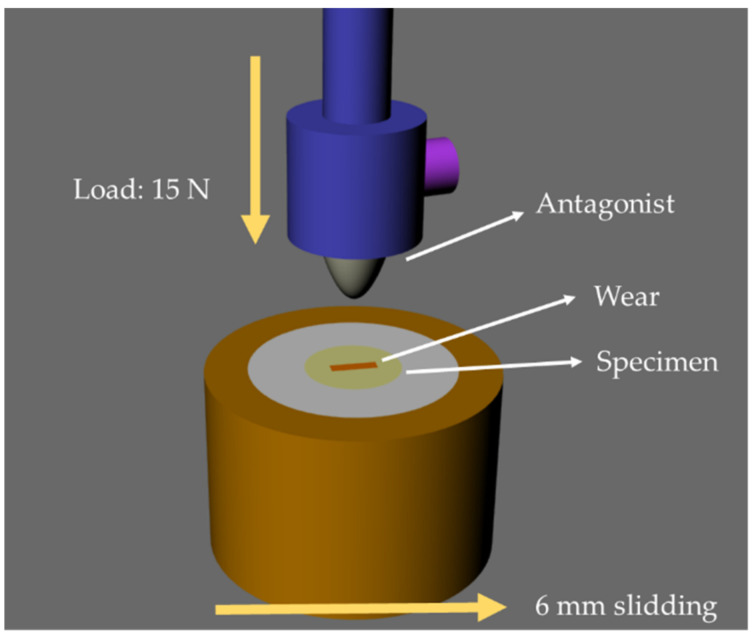
Descriptive illustration of the wear simulation test. The indenter reaches the specimen with a load of 15 N and slides 6 mm before returning to the initial position.

Physiological wear simulation on the top surfaces of the samples was performed in a sliding wear machine (Biocycle V2, Biopdi, São Carlos, São Paulo, Brazil). The wear was evaluated against two different antagonists: steatite (a magnesium silicate-based ceramic, whose wear behavior ranged between enamel and glass ceramics) (Chiarotti Ceramics, Jaguariúna, São Paulo, Brazil) and PICN (*n* = 10). Cylinder-shaped pistons with a round tip (6 mm Ø) were machined from each material. During the tests, a vertical load was applied onto the specimens’ surfaces followed by 6 mm horizontal sliding (Figure 1). The samples were subjected to loads of 15 N for 5000 cycles at 1.7 Hz of frequency. The tests were carried out in distilled water. The mass of the specimens and the antagonists were evaluated with an analytic scale (Adventurer-AR Analytical; Ohaus, Barueri, São Paulo, Brazil) at baseline and at the end of the study. The difference between the final and initial masses was determined as the mass loss during the wear simulation. The tests were paused at 10, 100, 500, and 1000 cycles for roughness measurements.

### 2.3. Roughness

The surface roughness of all the samples was measured at baseline and after 10, 100, 500, 1000, and 5000 cycles of wear simulation was assessed with a contact profilometer (Mitutoyo SJ-410; Mitutoyo Corporation, Takatsu-ku, Kawasaki, Kanagawa, Japan). Six measurements were performed: two at each of the 0°, 45°, and 90° angles. The Ra parameter (average roughness) and RSm (mean width of the profile elements) were obtained with a cut-off of 0.8 mm and a speed of 0.5 mm/s. The means (Ra and RSm) of the six measurements of each sample were obtained.

### 2.4. Scanning Electron Microscopy (SEM)

Representative specimens and antagonists (*n* = 1) from each experimental group were analyzed in a scanning electron microscope (SEM, Inspect S 50, FEI Company, Brno, Czech Republic). Topographic features before and after the wear simulation were observed in the ceramic discs and the antagonists.

### 2.5. Data Analysis

Mass loss and roughness data were tested for normality (Shapiro–Wilk test) and homoscedasticity (Levene’s test). Ra and RSm were analyzed by three-way ANOVA (surface treatment × glaze presence × antagonist), followed by Tukey’s test. Due to non-normal distribution and non-homoscedasticity, mass loss data were subjected to the Kruskal–Wallis test followed by the Man–Whitney test for two-on-two comparisons within each of the antagonists. The significance level was set at 5% for all statistical tests.

## 3. Results

### 3.1. Wear and Roughness Analyses

Surface treatment influenced the steatite antagonist mass loss during the physiological wear (*p* = 0.000). However, PICN pistons showed a similar mass loss regardless of the experimental group (*p* = 0.632) (Table 3). The average mass loss of steatite was 0.0027 ± 0.001 g and 0.0021 ± 0.0006 g for PICN.

Regarding the ceramic wear, different surface-etching treatments affected the mass loss against the steatite antagonist (*p* = 0.012). The highest mass loss was observed in the AG group. However, there was no statistically significant difference when PICN was used as an antagonist (*p* = 0.246) (Table 3).

**Figure 2:**
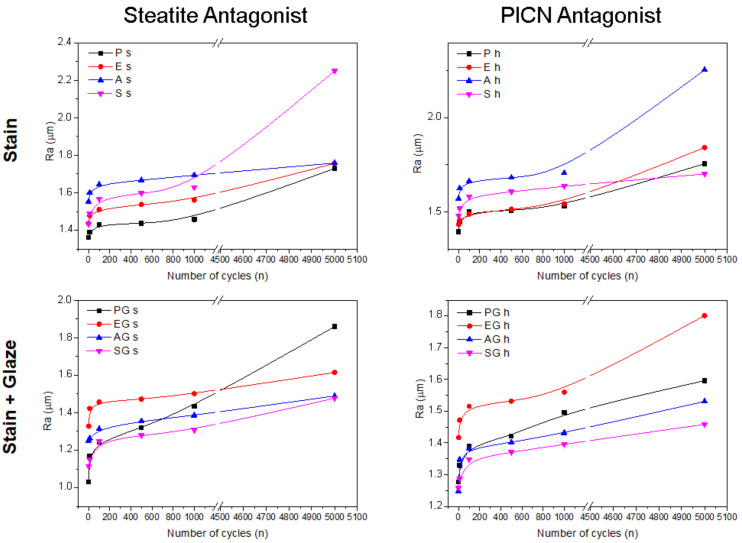
Line graphs showing the average roughness (Ra) of each experimental group at the established intervals.

**Figure 3:**
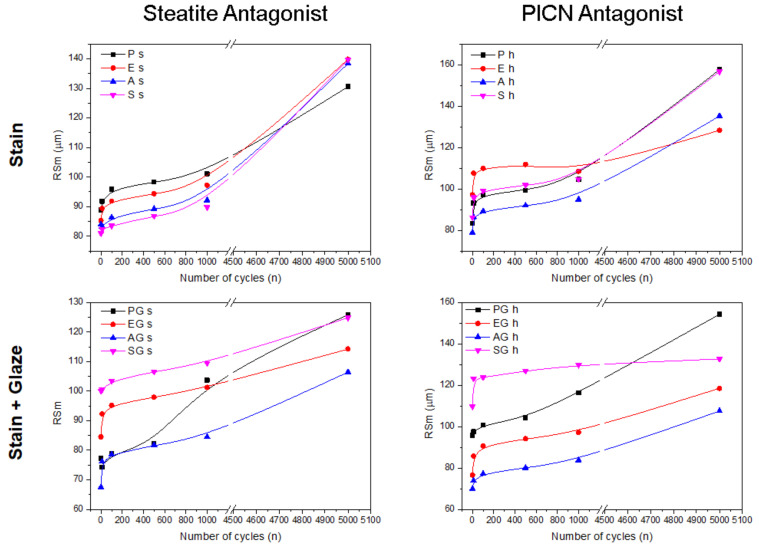
Line graphs showing the spacing roughness (Rsm) of each experimental group at the established intervals.

Figure 2 depicts line graphs of the mean roughness (Ra), and Figure 3 shows the spacing roughness (RSm) of the experimental groups during the physiological wear simulation at 10, 100, 500, 1000, and 5000 cycles.

Statistical analysis of Ra data showed a significant interaction among the factors of surface treatment × glaze presence × antagonist (*p* = 0.000). However, there was no statistically significant interaction among the three factors in Rm data (*p* = 0.234). Even so, RSm was affected by each individual factor: glaze (*p* = 0.005), antagonist (*p* = 0.000), and surface treatment (*p* = 0.000). Table 4 shows the means of the average roughness of each experimental test after the wear simulation under different antagonists.

The self-etching primer (S) resulted in the highest average roughness (0.82 ± 0.19 µm) against the steatite indenter. The polished (P) and acid-etched (E) samples showed similar average roughness under both steatite (P: 0.37 ± 0.17 µm, E: 0.32 ± 0.17 µm) and PICN (P: 0.36 ± 0.28 µm, E: 0.41 ± 0.24 µm) antagonists. The glaze application decreased the average roughness of all groups when compared to their counterparts (Table 4).

### 3.2. Scanning Electron Microscopy (SEM)

**Figure 4:**
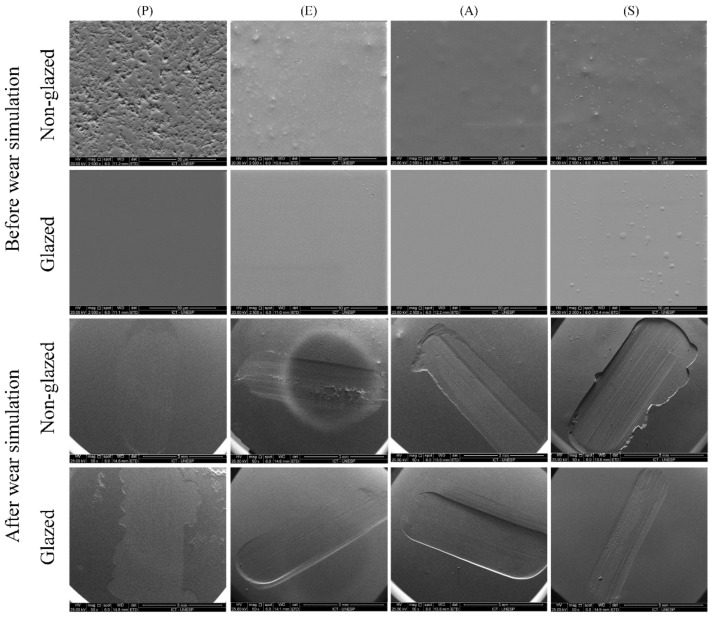
SEM images of the specimen surfaces after 5000 cycles of wear simulation at 50× magnification. Before wear simulation (2500×), only non-glazed P specimens showed irregular surfaces, whereas all groups, including glazed specimens, showed uniform surfaces. After wear simulation, wear craters were evidenced in all groups.

SEM topographic images of samples from each experimental group are presented in Figure 4. Similar features were observed on the samples tested with the steatite and PICN antagonists. Thus, representative specimens of each surface-etching treatment tested with any of the antagonists were selected for SEM inspection.

Smoother surfaces are observed in the glazed groups. Among the groups without glaze, the E, A, and S groups showed more homogeneous surfaces, whereas voids and grooves were observed on the surface of P (Figure 4). The AG group showed a more homogeneous surface, and the SG group showed build-up in clusters (Figure 4). After the physiological wear simulation, all groups showed similar wear craters and scratchers from the antagonist contact. As an exception, the P group showed less prominent wear craters, since no characterization layer was applied to its surface.

**Figure 5:**
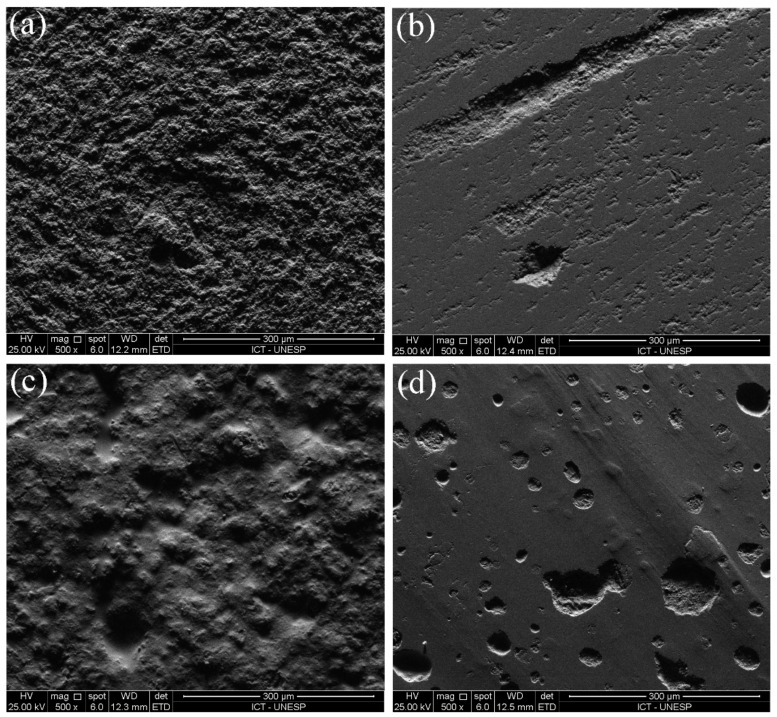
SEM images of PICN (**a**,**b**) and steatite antagonists surfaces (**c**,**d**) at 500× magnification, before (left) and after (right) the wear simulation. Uneven surfaces were observed before the tests, which turned more homogeneous after wear.

Figure 5 depicts the PICN (a,b) and steatite (c,d) antagonists before and after the wear simulation. Figure 5a,c show grooves from the milling process on both materials. After the wear simulation (b,d), bigger grooves and defects were evidenced on the worn-off area.

## 4. Discussion

The wear resistance of ceramic materials is paramount to the understanding of possible drawbacks in prosthetic rehabilitation treatment, since wear is an inevitable process [16,17]. In this study, we analyzed the wear behavior of the characterization layer of PICN subjected to different surface-etching treatments and antagonists. Our results pointed out that the roughness and mass loss of PICN is affected by the surface treatment and the antagonist. Thus, the tested hypothesis was rejected.

PICN showed similar wear behavior and higher Ra values when only the stain was applied (Groups E, A, and S). The EG, AG, and SG groups showed lower Ra values, since a glaze layer was applied after the stain. These results are in agreement with those from Kurt et al. (2019) [18] and Incesu, Yanikoglu (2019) [19] in which the authors reported that a glaze application in reinforced glass ceramics is the most effective way to reduce surface roughness.

The ceramic network of PICN allows a higher resistance to wear than do the traditional resin composites [20]. The glazed groups (EG, PG, and SG) showed higher wear resistance. Thus, the polymeric glaze layer had a protective effect on the PICN surfaces. These findings are in agreement with Tribst et al. (2020) [21], who reported that a glaze layer was promising in improving characterization durability in hybrid ceramics. Clinically, PICN wears off when in contact with enamel and other restorative materials antagonists [22,23]. The ceramic network is wear- and deformation-resistant, as well as fragile and susceptible to tensile fracture. On the other hand, the infiltrated polymeric matrix leads to plastic deformation and causes energy dissipation [24], which can improve the mechanical behavior under compressive loads [25].

The use of different antagonists did not result in significant Ra and RSm differences. Steatite presents an elastic modulus similar to that of enamel [26], whereas the elastic modulus of PICN is similar to that of dentin [27]. Therefore, as the elastic modulus of both materials lies in the range of those from dental tissues, similar wear and deformation was produced on the material surfaces. This also evidences the clinical appeal of the present study, since a common clinical situation was simulated.

In addition to the materials’ microstructure, the load and number of cycles influences wear. A previous study [22] applied a load of 49 N with 1.6 Hz of frequency for 120,000 cycles on PICN samples. Xu et al. (2017) [28] used a 20 N load at 2 Hz for 50,000 cycles on PICN. In the aforementioned studies evaluating the wear of the PICN samples, high numbers of cycles were used. Our study aimed to evaluate the removal of the characterization layer; a pilot study demonstrated that 5000 cycles was enough for this purpose. In addition, the tests were paused at established intervals (0, 10, 100, 500, 1000, and 5000) for better monitoring of the wear and roughness measurements. The stain and glaze indicated for finishing PICN restorations are polymeric materials, which explains the prominent wear after only 5000 cycles. Moreover, a previous study reported that the characterization layer of a hybrid ceramic has been completely removed after 200,000 cycles [29].

A 5% hydrofluoric acid etching for 60 s and sandblasting with 50 µm aluminum oxide are the treatments proposed by the manufacturer of PICN prior to the stain and glaze application [30]. In our study, the EG and AG groups reached the lowest roughness results after cycling with the steatite antagonist. According to Emsermann et al. (2019) [31], sandblasting the PICN surface with aluminum oxide is a viable alternative for improving the bond strength between the ceramic and the characterization layer. Tribst et al. (2020) [21] evaluated the three-body wear of hybrid ceramics and concluded that acid etching should be used to improve the longevity of the characterization layer, which is in agreement with our findings. Moreover, a previous study [4] has demonstrated that acid etching led to the best results in bond strength between PICN and staining. Apparently, the surface roughening caused by hydrofluoric acid allows better interlocking between ceramic and stain. This mechanism not only improves bond strength, but also makes it more difficult for the stain to wear off.

The self-etching primer led to wear results similar to those of the acid-etched groups. However, when tested against steatite, the S group showed a Ra mean value of 0.82 µm, whereas the other groups ranged between 0.24 and 0.37 µm. Tribst et al. (2020) [21] and Al-Harthi et al. (2018) [32] reported that a self-etching primer was a viable treatment for the durability of the characterization layer on PICN. Despite this, as of now, using a self-etching primer makes the treatment more expensive than etching with hydrofluoric acid, which must not be taken for granted.

Our study investigated the wear behavior of PICN after different surface treatments prior to the stain and glaze application. However, there is little to no evidence regarding the effect of these treatments on the mechanical behavior (e.g., survival, load to failure, flexural strength) of hybrid ceramics. Since similar wear results were observed among treatments (especially when tested with a PICN antagonist), mechanical properties studies are encouraged to enhance the literature on this topic and guide us toward the best treatments. Nevertheless, considering our surface and antagonist wear and roughness results, acid-etching the PICN surface prior to the stain and glaze application is advised, especially in areas subjected to two-body wear.

## 5. Conclusions

Hydrofluoric acid etching results in a combination of less surface wear and a smooth surface on hybrid ceramics characterized with a stain and glaze after two-body physiological wear simulation. The tested antagonists did not result in significant roughness differences on the PICN surface. The glaze application after staining is paramount to ensure a smoother surface and avoid antagonist wear.

## Figures and Tables

**Table 1:** Materials, brands, manufacturers, and chemical compositions of the materials used in the study.

Material	Brand	Manufacturer	Composition
Hybrid ceramic (PICN)	Vita Enamic	Vita Zahnfabrik	86w% feldspathic ceramic: SiO_2_ 58–63%, Al_2_O_3_ 20–23%, Na_2_O 9–11%, K_2_O 4–6%, 14w% polymer: TEGDMA,UDMA
Etching agent	Vita adiva^®^ cera-etch	Vita Zahnfabrik	5% hydrofluoridric acid
Ceramic primer	Vita adiva^®^ c-prime	Vita Zahnfabrik	Solution of methacrylsilanes in ethanol
Self-etching ceramic primer	Monobond Etch & Prime	Ivoclar Vivadent	Butanol, tetrabutylammonium dihydrogen trifluoride, methacrylated phosphoric acid ester, bis(triethoxysilyl)ethane, silane methacrylate, colourant, ethanol, water
Stain	Vita enamic^®^ stain	Vita Zahnfabrik	Cristobalite, dibenzoyl peroxide, dicyclohexyl phthalate
Stains liquid	Vita enamic^®^ stains liquid	Vita Zahnfabrik	methyl methacrylate, aromatic urethanacrylate
Glaze	Vita enamic^®^ glaze	Vita Zahnfabrik	methyl methacrylate, 2-Propenoic acid, reaction product with Pentaerythrite, Diphenyl (2,4,6-trimethylbenzoyl) phosphinoxide
Air spray: Al_2_O_3_	Aluminium oxide	Bio Art	Al_2_O_3_ 50 µm

**Table 2:** Experimental design.

Groups	Surface Treatments	Finishing
P	Polishing	-
PG		Glaze
E	5% hydrofluoric etching (E) for 60 s, cleaning (ultra-sonic bath with distilled water for 5 min), and silanization.	Stain
EG		Stain plus Glaze
A	Aluminum oxide (Al_2_O_3_) 50 μm at 1 bar sandblasting, cleaning (ultra-sonic bath with distilled water for 5 min), and silanization.	Stain
AG		Stain plus Glaze
S	Silanization with etch-prime adhesive (S) and cleaning (ultra-sonic bath with distilled water for 5 min).	Stain
SG		Stain plus Glaze

**Table 3:** Mass loss from the specimens (discs), the antagonists (pistons), and homogeneous groups according to the Mann–Whitney test.

	Mass Loss of the PIC Discs		Mass Loss of the Antagonist Piston	
	Steatite	PICN	Steatite	PICN
P	0.0029 ± 0.001 ^BC^	0.0030 ± 0.001	0.0014 ± 0.003 ^A^	0.0.001 ± 0.0001
PG	0.0018 ± 0.00 ^C^	0.0021 ± 0.001	0.0014 ± 0.001 ^B^	0.0001 ± 0.0001
E	0.0034 ± 0.001 ^BC^	0.0010 ± 0.000	0.0010 ± 0.001 ^D^	0.0001 ± 0.0001
EG	0.0020 ± 0.001 ^C^	0.0017 ± 0.000	0.0014 ± 0.003 ^A^	0.0001 ± 0.0001
A	0.0011 ± 0.000 ^C^	0.0026 ± 0.001	0.0005 ± 0.001 ^C^	0.0001 ± 0.0001
AG	0.0042 ± 0.002 ^A^	0.0024 ± 0.001	0.0001 ± 0.001 ^D^	0.0001 ± 0.0000
S	0.0036 ± 0.001 ^B^	0.0022 ± 0.001	0.0006 ± 0.001 ^C^	0.0001 ± 0.0000
SG	0.0029 ± 0.001 ^BC^	0.0023 ± 0.001	0.0004 ± 0.003 ^C^	0.0001 ± 0.0000

PICN discs and antagonists did not show statistically significant differences among the groups (*p* = 0.632 and *p* = 0.246, respectively). Different letters in steatite antagonist columns show the statistically significant differences among the groups.

**Table 4:** Means and standard deviations of average roughness (µm) of each experimental group at the end of the physiological wear simulation.

	Steatite	PICN
P	0.37 ± 0.17 ^Ba^	0.36 ± 0.28 ^Ba^
PG	0.36 ± 0.27 ^Aa^	0.32 ± 0.20 ^Ab^
E	0.32 ± 0.17 ^Ba^	0.41 ± 0.24 ^Ba^
EG	0.29 ± 0.08 ^Ba^	0.38 ± 0.26 ^Aa^
A	0.21 ± 0.02 ^Cb^	0.69 ± 0.43 ^Aa^
AG	0.24 ± 0.11 ^Ba^	0.28 ± 0.06 ^Aa^
S	0.82 ± 0.19 ^Aa^	0.24 ± 0.14 ^Bb^
SG	0.36 ± 0.12 ^Ba^	0.20 ± 0.08 ^Aa^

Different capital letters in the same line indicate a comparison between antagonists within the same surface-etching treatment. Lower-case letters in the same column indicate significant differences among the experimental groups within the same antagonist (Tukey’s test, *p* < 0.05).
